# A Sustainable Evaluation of Drilling Parameters for PEEK-GF30

**DOI:** 10.3390/ma6125907

**Published:** 2013-12-13

**Authors:** Rosario Domingo, Manuel García, Alberto Sánchez, Rosa Gómez

**Affiliations:** 1 Department of Construction and Manufacturing Engineering, Universidad Nacional de Educación a Distancia (UNED), C Juan del Rosal 12, Madrid 28040, Spain; E-Mail: rdomingo@ind.uned.es; 2 Department of Materials Science and Metallurgical Engineering, Graphic Expression in Engineering, Cartographic Engineering, Geodesy and Photogrammetry, Mechanical Engineering and Manufacturing Engineering, Universidad de Valladolid, P.º del Cauce 59, Valladolid 47011, Spain; E-Mail: asanchez@eii.uva.es; 3 Department of Chemistry Applied to Engineering, Universidad Nacional de Educación a Distancia (UNED), C Juan del Rosal 12, Madrid 28040, Spain; E-Mail: rgomez@ind.uned.es

**Keywords:** finish, forming, composite materials, sustainable process, PEEK-GF30

## Abstract

This paper presents a study of hole quality and energy consumption in the process of drilling a thermoplastic polymeric material, polyether-ether-ketone, reinforced with 30% glass fibers (PEEK-GF30). PEEK-GF30’s capacity to be machined has focused on turning operations. Studies of drilling involving thermoplastic polymeric materials have considered materials with other types of matrices, or reinforcement. In this study, quantities such as maximum and mean surface roughness, delamination, maximum thrust force, maximum momentum, and energy required during the process were determined for three types of drill bits, and the most influential factors for each variable were identified using an ANOVA multifactor analysis. The highest quality and lowest energy consumption were achieved for a drill bit rotation speed of 7000 rpm and a feed rate of 400 mm/min with a tungsten carbide (WC) drill bit coated with titanium aluminum nitride (TiAlN). Although a WC drill bit with a diamond point reduces delamination, the roughness increases, thus, the choice of the drill bit type depends on the roughness allowed. A WC drill bit coated with TiAlN produces a good surface finish that can eliminate subsequent operations and requires less energy; thus, this type of drill bit is the most attractive of the types evaluated.

## 1. Introduction

Polymeric materials that have organic matrices reinforced with glass fibers are increasingly being used in industry because of their mechanical characteristics. In fact, it is possible to find these materials in gears, pistons, structural components and exchange membranes [1]. The addition of glass fibers to the polymers results in materials with improved mechanical and thermal properties, allowing a wider range of applications. Among these types of materials, polyether-ether-ketone, reinforced with 30% glass fibers (PEEK-GF30, which consists of the thermoplastic polymer polyether-ether-ketone 30% reinforced with glass fiber as defined by the DIN EN 8.513 standard, has the best properties. A typical forming operation with these materials is material removal to produce holes as a preliminary step to the insertion of fasteners (e.g., rivets or screws). The difficulties involved in drilling these types of materials are such that alternative hole-forming methods have been investigated [2].

The use of PEEK-GF30 in structural components has led to the study of its capacity to be machined [3,4,5], but research has focused on turning operations rather than drilling. Studies of drilling involving thermoplastic polymeric materials have considered materials with other types of matrices, such as polyester [6,7,8] and epoxy [9,10,11], or materials with other types of reinforcement, such as carbon fibers [12,13]. These studies and the literature reviews related to drilling polymeric composite materials [14,15,16,17] have shown that damage in the form of delamination must be minimized. The most commonly used drill bits are those made from high-speed steel and solid cemented carbide, but it is necessary to find other types that generate better results. Damage to the material (measured in terms of delamination) and the production of roughness are the variables of greatest interest for evaluating the quality of drilled holes.

Energy consumption in manufacturing processes is being studied more frequently [18,19], including the drilling process [20]. The energy required is dependent on the thrust force and the momentum, and these variables have been found to affect the surface quality [11] and delamination [6,7,8,10] of other glass-fiber-reinforced composite materials.

This paper presents an analysis of the quality of drilled holes in PEEK-GF30 and the energy required to produce them using several types of drill bits. A bit that improves the hole quality is desirable as holes of high quality will not require subsequent finishing operations in high-performance applications.

## 2. Experimental Section

### 2.1. Test Material and Test Samples

The material used was PEEK-GF30, for which the most significant mechanical and thermal properties and a comparison with unreinforced PEEK are shown in Table 1 (the data were provided by the material supplier). It can be observed that the glass fiber reinforcement improves several properties of PEEK, which facilitates its use in structural components.

**Figure 1:**
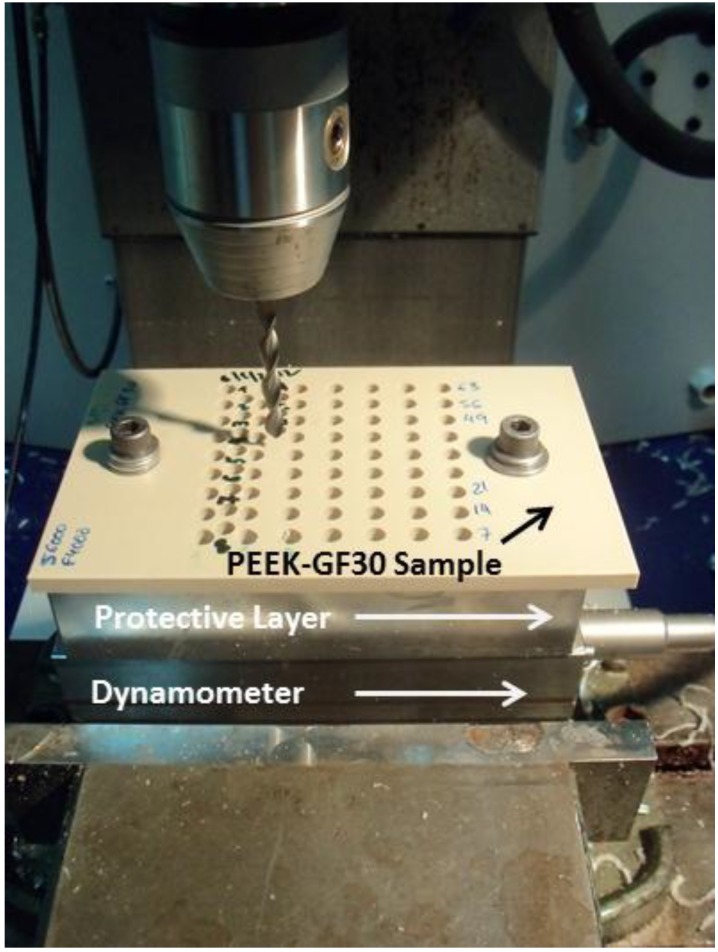
Piezoelectric dynamometer and test sample.

For the tests, 6.5-mm-thick plates of PEEK-GF30 were used. These plates were sized to fit the dynamometer used in the tests, which prevented buckling of the plates during the measurements of the forces and the momenta. To avoid damage to the equipment and the drill bits (see Figure 1) a protective layer was used between the plate and the dynamometer.

**Table 1:** PEEK and polyether-ether-ketone, reinforced with 30% glass fibers (PEEK-GF30) Properties.

Properties	PEEK GF30	PEEK
Density (kg/m^3^)	1490	1320
Hardness, Rockwell	M103	M99
Tensile Strength (MPa)	157	110
Tensile Modulus (GPa)	9.6995	4.482
Flexural Modulus (GPa)	10.309	4.14
Flexural Yield Strength (MPa)	233	179
Compressive Strength (MPa)	215	118
Shear Strength (MPa)	97.2	52.4
Point of Fusion (°C)	343	334
Heat capacity (J/(g·K))	0.43	0.32

### 2.2. Machine, Tool, and Process Parameters

Drilling tests were performed with drill bits of various materials and geometries: a standard high-speed steel (HSS) bit (bit *B*1); a cemented carbide (WC) bit with a TiAlN coating (bit *B*2), designed for universal use; and a WC bit with a diamond point (drill bit *B*3), designed specifically for use in composite and polymeric materials. Bit *B*1 had the lowest cost of the three, and bit *B*3 had the highest cost (approximately 40 times higher than *B*1 and 30 times higher than *B*2). All the bits had a diameter of 6.3 mm, and their characteristics are given in Table 2. The material properties and geometries of the types of drill bits tested can be found in the literature [9].

Drilling was performed using a Manga Tongtai TMV-510 machining center (Tong-tai Machine & Tool Co., Ltd, Kaohsiung Hsien, Taiwan,) with a FANUC controller (FANUC Corp., Oshino-mura, Japan). The following process parameters were used: drill bit rotation speeds (N) of 6000, 7000 and 8000 rpm and feed rates (F) of 300, 400 and 500 mm/min.

**Table 2:** Main data of drill bits for all tests.

Code	Material	Coated	Point Angle	Helix Angle	Web Thickness (mm)	Margin (mm)	Body Clearance (mm)
*B*1	HSS	–	130°	35°	1.25	0.43	0
*B*2	WC	TiAlN	140°	27°	1.9	0.40	0
*B*3	WC	Diamond Tip	90°	35°	2	0.22	0.16

### 2.3. Measurement of Force and Momentum and Evaluation of Energy Consumption

A Kistler 9257B piezoelectric dynamometer and a Kistler 5070A multichannel amplifier (Kistler Instrument Corp., Novi, MI, USA) were used to collect the thrust force and momentum data, which were measured with respect to the feed axis (*Z*-axis), and the energy required for each drill bit was determined from these quantities. The maximum force (*Fz*_max_) and the maximum momentum (*Mz*_max_) were used because these are required to produce the hole.

### 2.4. Evaluation of the Surface Quality and Delamination Factor

**Figure 2:**
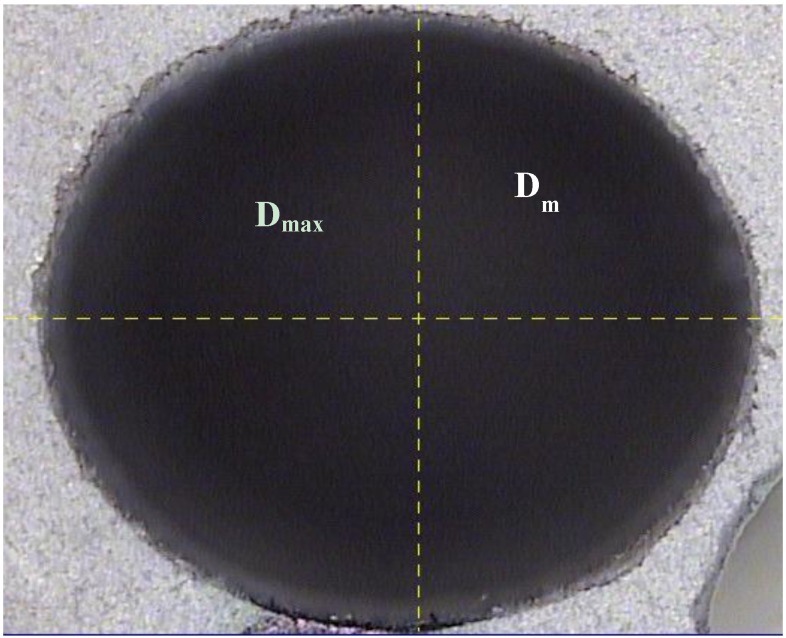
Hole example: nominal (*D*) and maximum diameter (*D*_max_).

Subsequent to the drilling, the quality of the holes was determined based on the surface roughness and the delamination factor. The roughness was measured with a Mitutoyo SJ-400 surface roughness tester (Mitutoyo Corp., Kawasaki-shi, Japan) to obtain the values of maximum roughness (*R*_max_) and mean roughness (*R*_a_). The delamination factor was obtained using a three-dimensional measurement device with a TESA VISIO optical sensor (TESA SA, Renens, Switzerland) to measure the diameter (*D*) and the diameter of the damaged region (*D*_max_), from which the delamination factor (*Fd*), defined as the ratio *D*_max_/*D* [7,8], was calculated (see Figure 2). The use of this index allows comparison between values obtained with drill bits of different diameters. This ratio presents satisfactory results when delamination possesses a regular pattern, as in Glass Fiber Reinforced Plastic [21]. Davim* et al.* define ratio (the adjusted delamination factor) when delamination presents an irregular form [22].

### 2.5. Statistical Analysis

For each cutting condition and drill bit type, the test was repeated three times. The results of the tests were subjected to an analysis of variance (ANOVA) to determine whether there were significant differences at the 95% level of confidence; the analysis was conducted using the Statgraphics software [23]. The Fischer-Snedecor test uses the *F*-ratio and the *P*-value: a *P*-value greater than 0.05 implies that there are no significant differences between the means of the two sets of data [24]. This analysis was performed for the measurements of the maximum roughness (*R*_max_), the mean roughness (*R*_a_), the delamination factor, the feed force (*Fd*), the momentum (*Mz*_max_) and the energy (*E*) to determine which of the factors—rotation speed (*N*), feed rate (*F*), and drill bit type (*B*)—and their interactions (*N*-*F*, *N*-*B*, and *F*-*B*) were significant in the results. Although in the definition of *F* is implicated *N*, note that the interaction *N*-*F* allows determining the influence of a factor respect to the level of the other factor [24]; in fact, this interaction has been taken in account in the drilling [7].

## 3. Results and Discussion

As was previously indicated, the experiments were performed three times for each drill bit type and cutting condition to guarantee the precision of the measurements. Table 3 shows the resulting mean for each variable and cutting condition; the data were collected randomly, as can be observed in Table 3, to guarantee the independence of the results. These data were subjected to an ANOVA multifactor analysis. This analysis shows whether the factors *N*, *F*, and *B* and their interactions *N*-*F*, *N*-*B*, and *F*-*B* significantly impact the values of the variables *R*_max_, *R*_a_, *Fd*, *Fz*_max_, *Mz*_max_, and *E*. In addition, the error values (other sources of residual variation) and the corrected total (i.e., considering the error in the analysis) are shown. Table 4, Table 5, Table 6, Table 7 and Table 8 show the values of the sum of squares (*SS*), the degrees of freedom (*DF*), the root mean square (*RMS*), the *F*-ratio, the *P*-value, and the contribution, in percentages, of each factor and interaction to the results.

**Table 3:** Results summary.

Test Number	*N* (rpm)	*F* (mm/min)	*B*	*R*_a_ (μm)	*R*_max_ (μm)	*Fd*	*Fz*_max_ (N)	*Mz*_max_ (Nm)	*E* (J)
1	7000	300	3	2.18	16	1.08	88.92	0.89	407.43
2	7000	500	1	0.96	8.57	1.055	360.24	1.45	563.5
3	6000	500	3	0.67	6.12	1.033	66.348	0.548	227.52
4	6000	400	3	0.65	5.69	1.017	97.78	0.54	166.87
5	7000	400	3	0.92	8.07	1.016	78.25	0.45	244.13
6	6000	400	1	1.31	12.35	1.02	307.59	1.76	645.97
7	8000	300	1	1.13	8.98	1.052	263.56	0.79	573.92
8	6000	300	3	0.70	8.62	1.012	86.92	1.01	241.61
9	8000	500	2	0.97	6.75	1.051	173.27	0.57	183.48
10	8000	400	2	0.64	5.16	1.025	125.72	1.31	513.01
11	6000	500	1	3.16	25.62	1.085	559.65	0.82	283.37
12	7000	400	1	1.23	10.53	1.037	315.3	1.43	773.77
13	8000	500	3	5.46	46.42	1.078	74.71	0.56	174.82
14	7000	400	2	0.55	5.17	1.049	154.82	0.43	151.72
15	7000	300	2	1.03	12.65	1.033	148.55	0.66	139.62
16	7000	300	1	1.56	11.51	1.043	251.43	1.24	956.37
17	6000	300	2	0.6	8.44	1.020	174.87	0.69	441.39
18	8000	300	2	0.50	4.25	1.063	119.18	0.64	436.06
19	8000	300	3	2.06	7.21	1.106	80.5	0.28	131.19
20	7000	500	3	0.60	5.18	1.021	78.55	0.51	298.79
21	7000	500	2	0.50	5.03	1.056	157.72	0.74	217.56
22	8000	400	1	1.41	11.91	1.050	321.35	0.76	371.2
23	6000	500	2	0.97	8.24	1.053	178.40	0.62	316.15
24	8000	500	1	3.47	29.02	1.086	537.53	0.92	425.28
25	8000	400	3	1.09	9.27	1.011	65.99	0.74	444.9
26	6000	300	1	1.13	11.75	1.042	241.66	1.56	418.62
27	6000	400	2	0.72	5.84	1.028	187.42	0.83	268.06

**Table 4:** *Fz*_max_ analysis of variance.

*Fz*_max_	*SS*	*DF*	*RMS*	*F*-Ratio	*P*-Value	Contribution (%)
*N*	11,877.2	2	5,938.62	10.05	0.0002	0.84
*F*	95,201.4	2	47,600.7	80.53	<0.0001	6.77
*B*	1,052,230	2	526,115.0	890.10	<0.0001	74.85
*N–F*	18,577.4	4	4,644.36	7.86	<0.0001	1.32
*N–B*	20,191.5	4	5,047.87	8.54	<0.0001	1.44
*F–B*	170,971.0	4	42,742.8	72.31	<0.0001	12.16
Error	36,646.7	62	591.076	–	–	2.61
Corrected Total	1,405,700	80	–	–	–	–

**Table 5:** *Mz*_max_ analysis of variance.

*Mz*_max_	*SS*	*DF*	*RMC*	*F*-Ratio	*P*-Value	Contribution (%)
*N*	0.5677	2	0.2838	7.75	0.0010	4.86
*F*	0.4028	2	0.2014	5.50	0.0063	3.45
*B*	5.0963	2	2.5481	69.59	<0.0001	43.67
*N–F*	1.3567	4	0.3392	9.26	<0.0001	11.63
*N–B*	1.6521	4	0.4130	11.28	<0.0001	14.16
*F–B*	0.3234	4	0.0808	2.21	0.0784	2.77
Error	2.2702	62	0.0366	–	–	19.45
Corrected Total	11.6692	80	–	–	–	–

**Table 6:** *E* analysis of variance.

*E*	*SS*	*DF*	*RMC*	*F*-Ratio	*P*-Value	Contribution (%)
*N*	95,698.6	2	47,849.3	2.59	0.0832	2.51
*F*	214,772.0	2	107,386.0	5.81	0.0049	5.63
*B*	1,418,000	2	709,001.0	38.36	<0.0001	37.17
*N–F*	85,216.0	4	21,304.0	1.15	0.3404	2.23
*N–B*	759,763.0	4	189,941.0	10.28	<0.0001	19.91
*F–B*	95,754.5	4	23,938.6	1.30	0.2817	2.51
Error	1,145,980	62	18,483.5	–	–	30.04
Corrected Total	3,815,190	80	–	–	–	–

**Table 7:** *R*_max_ and *R*_a_ analysis of variance.

***R*_max_**	***SS***	***DF***	***RMC***	***F*-Ratio**	***P*-Value**	**Contribution (%)**
*N*	394.776	2	197.388	10.31	0.0001	6.09
*F*	819.937	2	409.969	21.42	<0.0001	12.64
*B*	848.94	2	424.47	22.18	<0.0001	13.09
*N–F*	1888.96	4	472.239	24.68	<0.0001	29.12
*N–B*	883.994	4	220.998	11.55	<0.0001	13.63
*F–B*	464.473	4	116.118	6.07	0.0003	7.16
Error	1186.53	62	19.1375	–	–	18.29
Corrected Total	6487.6	80	–	–	–	–
***R*_a_**	***SS***	***DF***	***RMC***	***F*-Ratio**	***P*-Value**	**Contribution (%)**
*N*	10.9892	2	5.4946	23.57	<0.0001	11.25
*F*	12.0282	2	6.0141	25.79	<0.0001	12.32
*B*	15.6089	2	7.8044	33.47	<0.0001	15.99
*N–F*	23.4433	4	5.8608	25.14	<0.0001	24.01
*N–B*	15.4628	4	3.8657	16.58	<0.0001	15.84
*F–B*	5.6513	4	1.4128	6.06	<0.0004	5.79
Error	14.456	62	0.233161	–	–	14.81
Corrected Total	97.6397	80	–	–	–	–

**Table 8:** *Fd* Analysis of Variance.

*Fd*	*SS*	*DF*	*RMC*	*F*-Ratio	*P*-Value	Contribution (%)
*N*	0.0077	2	0.0038	13.09	<0.0001	12.75
*F*	0.0125	2	0.0063	21.30	<0.0001	20.7
*B*	0.0019	2	0.0009	3.29	0.0438	3.15
*N–F*	0.0074	4	0.0018	6.31	0.0003	12.25
*N–B*	0.0038	4	0.0009	3.22	0.0182	6.29
*F–B*	0.0087	4	0.0022	7.41	0.0001	14.4
Error	0.0182	62	0.0003	–	–	30.13
Corrected Total	0.0604	80	–	–	–	–

### 3.1. Thrust Force

The ANOVA analysis (Table 4) shows that all of the factors and their interactions are significant in the determination of *Fz*_max_. However, the degree of influence is quite diverse, being much higher for the drill bit type, followed by the *F*–*B* interaction, which together accounted for 87%. The interaction *N*–*F* has a low contribution respect to *F* because the thrust force is strongly dependent on the feed rate. Thus, the thrust force is little influenced by levels of N.

**Figure 3:**
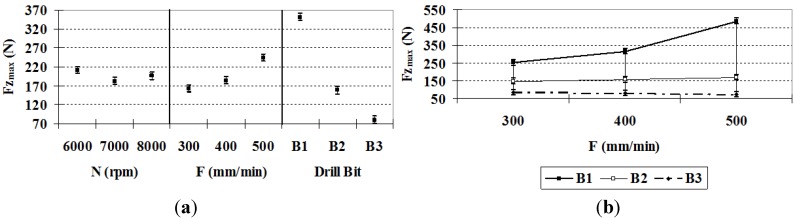
(**a**) *Fz*_max_ (95% confidence interval); (**b**) *Fz*_max_ *F*–*B* interactions (95% confidence interval).

It can be observed in Figure 3a that *Fz*_max_ is lower for drill bit *B*2 and lowest for drill bit *B*3. This result implies that the specific design of drill bit *B*3 achieves a good incidence in this type of material without notable differences in the cutting conditions. It is noteworthy that the *B*3 drill bit has a point angle of 90°, and it is made of a harder material than *B*2, which is harder than *B*1, implying that *B*3 undergoes less deformation during the drilling process. Regarding the next decisive factor, the *F*–*B* interaction, the behavior of the variable depends on the variation of *F* with respect to the drill bit type (see Figure 3b). With drill bit *B*3, the forces remain approximately constant as *F* increases; thus, the use of higher values, which reduces the manufacturing time, is recommended.

Finally, it should be noted that the cutting speed has a negligible influence on *Fz*_max_ [12].

Theses results are in agreement with what Abrão* et al.* have found when drilling glass fiber reinforced plastic composite [25].

### 3.2. Momentum

In the analysis of momentum (see Table 5), it was found that all of the factors and interactions are significant at a 95.0% level of confidence, except for the *F*–*B* interaction. It was also observed that nearly 70% of the variance is attributable to *B* and the interactions *N*–*B* and *N*–*F*.

**Figure 4:**
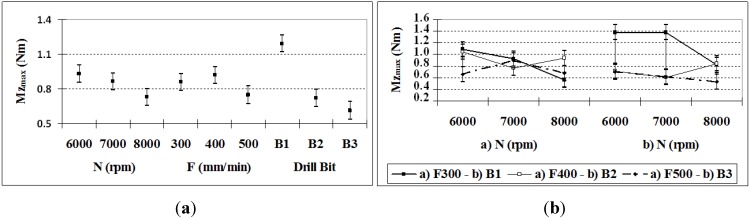
(**a**) *Mz*_max_ (95% confidence interval); (**b**) *Mz*_max_ *N*–*F* and *F*–*B* interactions (95% confidence interval).

The momentum does not present a regular pattern, but it can be observed that the *B*1 drill bit gives higher values, and the *B*3 drill bit gives lower values (Figure 4a), which can be explained by the same causes as the feed force results.

Regarding the interaction (Figure 4b) *N*–*B*, when the rotation speed increases for drill bit *B*3, the momentum decreases, whereas in relation to *N*–*F*, the lower feed rate results in a greater reduction in the momentum at high speeds, as expected because of the concept of the feed rate.

In the case of *Mz*_max_, it is observed that the rotation speed of the drill bit is more relevant (except for the significant effect of the drill bit type), unlike *Fz*_max_, in which the feed rate is predominant.

### 3.3. Energy

The analysis of the energy consumed in drilling requires knowledge of the thrust force and the momentum because both variables contribute to the energy. Table 6 shows that only, *B*, *F* and the *N*–*B* interaction are significant because, for the others, the *P*-value is greater than 0.05. Of the three, the variable that has the greatest influence is the drill bit type, followed by the *N-B* interaction and the feed rate, which together account for nearly 63% of the variance. It can be observed that the first two variables are the same variables that have the most influence on the momentum, which is expected because the momentum is usually responsible for more than 98% of the energy required [20].

**Figure 5:**
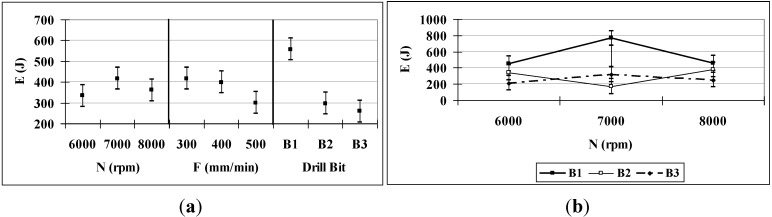
(**a**) *E* (95% confidence interval); (**b**) *E* *N*–*B* interaction (95% confidence interval).

Consistent with the results for the thrust force and the momentum, the drill bit type that results in the lowest energy consumption is *B*3 (see Figure 5b). As the feed increases, the energy consumption decreases (Figure 5a). The influence of N depends on the drill bit type: for *B*1 and *B*3, the energy decreases with increasing *N*, but in the case of *B*2 the energy does not follow the same pattern and the behavior is irregular (Figure 5b). The interaction *N*–*F* is not significant, so the influence of *N* on the level of *F* is negligible, mainly respect to the influence of the drill.

It will be shown in the following that similar behavior was observed in the mean and maximum roughness as in the momentum and the energy. It was observed that a larger amount of energy was consumed for the highest rotational speed of the drill bit (8000 rpm), with results very similar to those obtained for drill bits *B*2 and *B*3.

### 3.4. Surface Quality: Maximum and Mean Roughness

The surface quality was determined through the maximum and mean roughness, which were measured on the walls of the drilled holes and in the feed direction.

**Figure 6:**
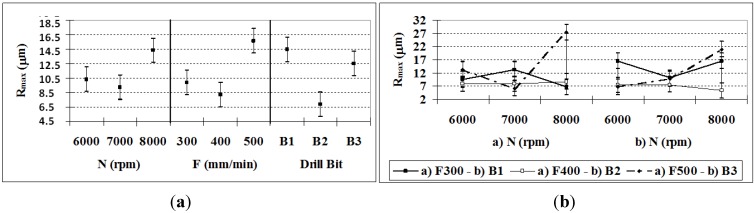
(**a**)* R*_max_ means and confidence intervals at 95.0%; (**b**) R_max_: *N*–*F* and *N*–*B* Interactions, and confidence intervals at 95.0% of *R*_max_.

According to the ANOVA (Table 7), all of the factors and their interactions have a significant effect on *R*_max_. Among them, the *N*–*F* and *N*–*B* interactions and *B* and *F* account for nearly 70% of the influence in the determination of *R*_max_. In Figure 6a, it can be observed that drill bit *B*2 and the feed rate *F* = 400 mm/min give the lowest *R*_max_ values. There is a direct relationship between the combinations of the factors *N*–*F* and *N*–*B* and *R*_max_; *R*_max_ decreases as the feed rate and the rotation speed decrease. This follows the concept of *F*, where *N* is already considered. Moreover, the drill bit type has a smaller influence for this variable (Figure 6b).

The *R*_max_ value is lower for the holes drilled with bit *B*2, which generally produces lower values for all of the cutting conditions, although *B*2 is better for lower feed rates. In this sense, the TiAlN coating is recommended for this material, especially when high cutting speeds are required, in mass production, and where a lower tool cost is desired.

**Figure 7:**
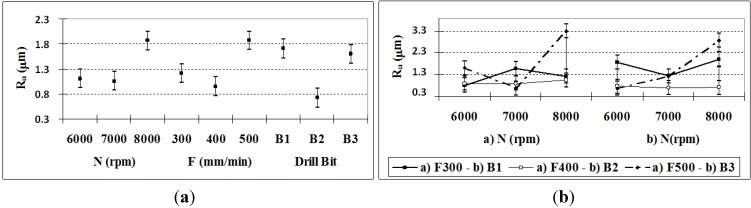
(**a**) *R*_a_ (95% confidence interval); (**b**) *R*_a_ *N–F* and *F–B* interactions (95% confidence interval).

In Table 7, it can be observed that all of the P-values for the factors and their interactions are lower than 0.05, so they all have significant effects on R_a_. However, the most influential factors on *R*_a_ are, in order of relevance, *N–F*, *B*, *N–B*, *F*, *N*, and *F–B*, where the first five account for 80%. In agreement with other studies [12,26] when *F* and *N* are increased, R_a_ should increase, but there are exceptions for *F* = 400 mm/min and *N* = 7000 rpm (see Figure 7a), which motivates the analysis of the interactions. These interactions have an incremental effect on *R*_a_ (Figure 7b), with the aforementioned exception (particularly for bit *B*2).

The maximum and mean roughness values show a close correlation. Table 7 indicates that the factors that contribute the most to both roughness measures are nearly the same. The factor with the greatest influence is the *N-F* interaction, followed by the drill bit type and the *N–B* interaction.

The mean roughness values produced by bits *B*2 and *B*3 were 0.5 and 0.6 μm, respectively, which implies a surface finish grade of N6 according to the ISO 1302 standard [27]. For bit *B*1, the minimum value of *R*_a_ was 0.96 μm, which equates to a grade of N7. In exterior turning operations with this material [3,4], higher mean roughness values were observed. Type *B*2 drill bits normally produce better surface quality (see Table 3), so the fact that bit *B*2 had the lowest mean roughness in the tests indicates that the conditions were favorable. Another possible explanation is that drilling generates higher temperatures than those of exterior turning operations, which has a favorable impact on the behavior of PEEK GF30 matrix material. The similar variations in *R*_max_ and *R*_a_ allow the establishment of a relationship between them, so that in future investigations, it will not be necessary to measure both to draw conclusions about the behavior of the tool in drilling operations with PEEK-GF30.

### 3.5. Delamination Factor

Regarding the delamination factor *Fd*, the ANOVA indicates that all of the factors and their interactions are significant and that the most influential factor is *F*, followed by *F–B*, and then *N* and *N-F*, which together account for more than 60% (Table 8). Theses results are in agreement with what other researchers have found when drilling reinforced polyester composites [7], glass fiber reinforced plastic composite [25], carbon fiber reinforced thermosets [28], or carbon fiber-reinforced plastic [29], and in opposition with what, Rubio* et al.* [8] have found when high speed drilling glass fiber reinforced plastic

**Figure 8:**
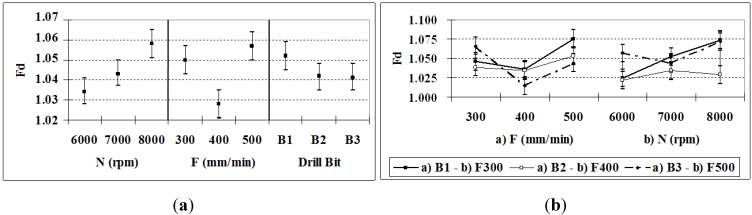
(**a**) *Fd* (95% confidence interval); (**b**) *Fd* *N–B* and *F–B* interactions (95% confidence interval).

The mean values of the delamination factor are shown in Figure 8a. Although increasing feed rate tends to increase the delamination, Figure 8b shows that Fd decreases for *F* = 400 mm/min, whereas an increase in *N* increases the delamination factor. In general, the combination of *F* and *B* is associated with less delamination up to the value *F* = 400 mm/min; bit *B*1 had worse behavior for *F* = 300 mm/min (Figure 8). It can be observed that the factors influencing *Fd* are very different from those influencing the surface quality.

The results obtained in all of the cases can be considered acceptable because the delamination factor was smaller than what has been observed in drilling similar materials, such as polyester reinforced with glass fibers [7]. The delamination factor was between 1.011 (*N* = 8000 rpm and *F* = 400 mm/min with drill bit *B*3) and 1.086 (*N* = 8000 rpm and *F* = 500 mm/min with drill bit *B*1). In general terms, higher rotation speeds, lower feed rates (so, low feed per revolutions), and smaller angles in the drill bit head (drill bit *B*3) reduce delamination. This result is consistent with those obtained using glass-fiber-reinforced epoxy [9] and, thus, indicate the best cutting conditions for the drilling of polymeric materials reinforced with glass fibers. Images of the drilled holes and the hole profiles, taken at 20× magnification, are provided in Table 9.

**Table 9:** Real hole and screen shoot from three-dimensional measurement device with a TESA VISIO optical sensor (PEEK G30 material).

Drill Bit	*N* (rpm)	*F* = 300 mm/min	*F* = 400 mm/min	*F* = 500 mm/min
*B*1	6000	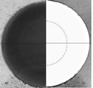	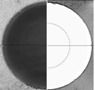	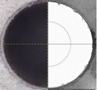
	7000	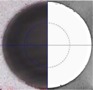	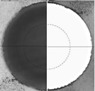	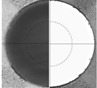
	8000	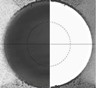	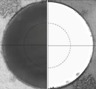	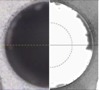
*B*2	6000	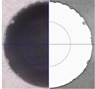	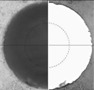	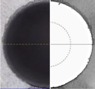
	7000	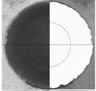	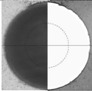	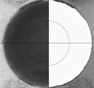
	8000	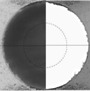	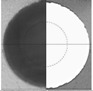	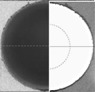
*B*3	6000	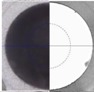	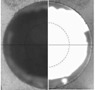	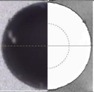
	7000	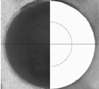	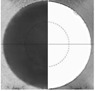	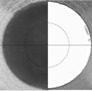
	8000	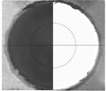	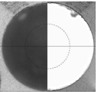	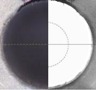

## 4. Conclusions

The results of an ANOVA multifactor analysis to determine the most influential factors on hole quality and energy consumption when drilling PEEK-GF30 was analysed. This analysis provides an enhanced look at the interactions of different influential factors.

In drilling tests performed with plates of PEEK-GF30, for the range of cutting conditions tested, the highest-quality holes were obtained for *N* = 7000 rpm, *F* = 400 mm/min and with drill bit *B*2. Drill bit *B*3 produced a lower delamination factor, but the surface roughness was greater, so the choice of the drill bit type will depend on the allowable roughness level (N6 or N7). The good surface finish obtained (which can make subsequent operations unnecessary) and the lower energy consumption make the *B*2 drill bit type the most attractive. In addition, the following observations can be made:

- The surface quality mainly depends on the relationship between *N* and *F*, where values higher than *N* = 7000 rpm and *F* = 400 mm/min, are not recommended. It was found that the maximum roughness was approximately 8.5 times higher than the mean roughness.
- The similar variations in *R*_max_ and *R*_a_ allow the establishment of a relationship between them, so that in future investigations, it will not be necessary to measure both to draw conclusions about the behavior of the tool in drilling operations with PEEK-GF30
- The delamination factor mainly depends on *F* (in agreement with others drilling composites studies), followed by the combination of *F* and *B*, but the drill bit type did not significantly affect *Fd*.
- The maximum thrust force depends largely on the drill bit type, with *B*3 requiring the least force, followed by *B*2. Increasing the feed rate resulted in an increase in the force, but the force was lower for drill bits *B*2 and *B*3.
- The type of drill bit is the most influential factor for the momentum, followed by the interactions *N-B* and *N-F*.
- The type of drill bit, the *N-B* interaction and the feed rate are the only significant factors in the energy consumed. The first two factors are the most relevant in the calculation of the momentum, which is the variable of greatest significance in the calculation of the energy.

The material PEEK-GF30 has been found suitable for drilling operations, which is favorable for high-productivity conditions in industrial manufacturing. In future work, a model for this material that can predict drilling quality as a function of the feed force and momentum and that can be used for other glass-fiber-reinforced materials and other reinforcement percentages will be investigated.
